# Elucidation of a novel *Vibrio cholerae* lipid A secondary hydroxy-acyltransferase and its role in innate immune recognition

**DOI:** 10.1111/j.1365-2958.2011.07765.x

**Published:** 2011-07-18

**Authors:** Jessica V Hankins, James A Madsen, David K Giles, Brandon M Childers, Karl E Klose, Jennifer S Brodbelt, M Stephen Trent

**Affiliations:** 1 Department of Biochemistry and Molecular Biology, Georgia Health Sciences UniversityAugusta, GA 30912, USA; 2 Department of Chemistry and Biochemistry, The University of Texas at AustinAustin, TX 78712, USA; 3 Section of Molecular Genetics and Microbiology, The University of Texas at AustinAustin, TX 78712, USA; 4 The Institute of Cellular and Molecular Biology, The University of Texas at AustinAustin, TX 78712, USA; 5 South Texas Center for Emerging Infectious Diseases and Department of Biology, The University of Texas at San AntonioSan Antonio, TX 78249, USA

## Abstract

Similar to most Gram-negative bacteria, the outer leaflet of the outer membrane of *Vibrio cholerae* is comprised of lipopolysaccharide. Previous reports have proposed that *V. cholerae* serogroups O1 and O139 synthesize structurally different lipid A domains, which anchor lipopolysaccharide within the outer membrane. In the current study, intact lipid A species of *V. cholerae* O1 and O139 were analysed by mass spectrometry. We demonstrate that *V. cholerae* serogroups associated with human disease synthesize a similar asymmetrical hexa-acylated lipid A species, bearing a myristate (C14:0) and 3-hydroxylaurate (3-OH C12:0) at the 2′- and 3′-positions respectively. A previous report from our laboratory characterized the *V. cholerae* LpxL homologue Vc0213, which transfers a C14:0 to the 2′-position of the glucosamine disaccharide. Our current findings identify *V. cholerae* Vc0212 as a novel lipid A secondary hydroxy-acyltransferase, termed LpxN, responsible for transferring the 3-hydroxylaurate (3-OH C12:0) to the *V. cholerae* lipid A domain. Importantly, the presence of a 3-hydroxyl group on the 3′-linked secondary acyl chain was found to promote antimicrobial peptide resistance in *V. cholerae*; however, this functional group was not required for activation of the innate immune response.

## Introduction

The Gram-negative bacterium *Vibrio cholerae* is the causative agent of the acute diarrheal disease cholera. Cholera is caused by the ingestion of contaminated food or water, causing rapid dehydration, and ultimately resulting in death when left untreated. *V. cholerae* thrive in marine environments and are readily found in temperate ocean waters. Within its aquatic environment more than 200 serogroups of *V. cholerae* have been identified; however, only *V. cholerae* strains bearing the lipopolysaccharide (LPS) somatic antigens O1 or O139 have been associated with pandemic disease (Mooi and Bik, 1997).

Lipopolysaccharide is the major constituent in the Gram-negative outer membrane and is composed of three regions: the lipid A domain, the core oligosaccharide and the O-antigen polysaccharide (Raetz and Whitfield, 2002). The core oligosaccharide consists of inner and outer core regions. The inner core is composed of 3-deoxy-D-*manno*-octulosonic acid (Kdo) residues, which link the lipid A domain to the remainder of the core oligosaccharide and O-antigen. Lipid A is responsible for anchoring LPS within the outer membrane of Gram-negative bacteria. Importantly, the lipid A domain is the bioactive portion of LPS and stimulates the host innate immune system through the activation of Toll-like receptor 4/myeloid differentiation factor 2 (TLR4/MD-2). Lipid A biosynthesis is a well-conserved and ordered process among Gram-negative bacteria (Raetz and Whitfield, 2002; Trent *et al*., 2006; Raetz *et al*., 2007). Despite the preservation of the Kdo-lipid A biosynthetic pathway, many diverse lipid A structures are produced by various pathogens, promoting survival during infection (Trent *et al*., 2006).

**Fig. 1:**
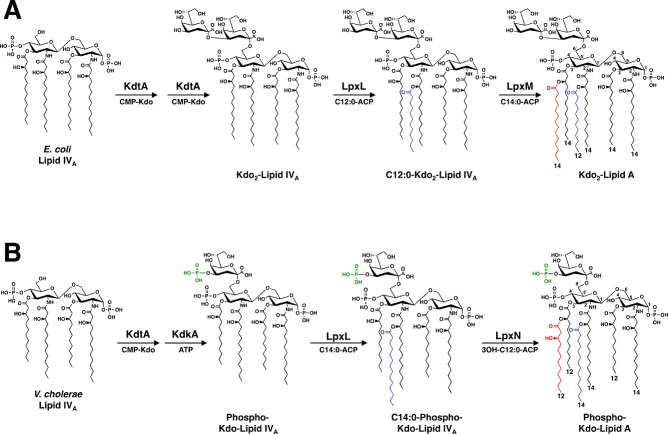
Comparison of the late stages of Kdo-lipid A biosynthesis in *E. coli* and *V. cholerae*. A. *E. coli* possess a bi-functional Kdo transferase (KdtA, also known as WaaA), which transfers two Kdo sugars to the lipid A precursor lipid IV_A_. The lipid A secondary acyltransferases, LpxL and LpxM then catalyse the transfer of a laurate (C12:0) (blue) to the 2′-position and a myristate (C14:0) (red) to the 3′-position of the glucosamine disaccharide, creating Kdo_2_-lipid A. B. The *V. cholerae* genome encodes a mono-functional KdtA, which transfers one Kdo residue to lipid IV_A_. The Kdo kinase (KdkA) then phosphorylates the Kdo sugar (green), resulting in phosphorylated Kdo-lipid IV_A_. *V. cholerae* LpxL (Vc0213) catalyses the transfer of a myristate (C14:0) (blue) to the 2′-position of the glucosamine disaccharide. As shown in the current work, LpxN (Vc0212) transfers a 3-hydroxylaurate (3-OH C12:0) (red) to the 3′-position, generating the hexa-acylated *V. cholerae* Kdo-lipid A domain. The exact order in which the secondary acyl chains are added in *V. cholerae* has not been determined.

Kdo-lipid A synthesis in *Escherichia coli* results in a hexa-acylated β-1′,6-linked disaccharide of glucosamine with unmodified phosphate groups at the 1- and 4′-positions (Fig. 1A) (Raetz and Whitfield, 2002). During the late stages of *E. coli* Kdo-lipid A biosynthesis, the bi-functional Kdo transferase KdtA (also known as WaaA) catalyses the transfer of two Kdo residues to the lipid A precursor, lipid IV_A_, creating Kdo_2_-lipid IV_A_ (Fig. 1A) (Clementz and Raetz, 1991). The addition of the Kdo residues is essential for the activity of the secondary acyltransferases LpxL and LpxM. LpxL and LpxM catalyse the transfer of laurate (C12:0) to the 2′-position (Clementz *et al*., 1996) and myristate (C14:0) to the 3′-position (Clementz *et al*., 1997) of the Kdo_2_-lipid IV_A_ precursor, respectively, creating Kdo_2_-lipid A.

Previously, little attention has been given to Kdo-lipid A biosynthesis in *V. cholerae*. The fatty acyl compositions of LPS isolated from various *V. cholerae* serogroups and biotypes have been characterized (Raziuddin and Kawasaki, 1976; Raziuddin, 1977; Broady *et al*., 1981; Kabir, 1982); however, a definitive Kdo-lipid A structure remains unknown. Our laboratory has previously shown that the late stages of *V. cholerae* Kdo-lipid A biosynthesis differ from that of *E. coli* (Hankins and Trent, 2009). The *V. cholerae* genome encodes a mono-functional KdtA, which catalyses the transfer of one Kdo residue to lipid IV_A_ (Fig. 1B). The *V. cholerae* Kdo kinase (KdkA) phosphorylates the Kdo residue, yielding phosphorylated Kdo-lipid IV_A_. The transfer of the phosphate group by KdkA is essential for the functionality of the *V. cholerae* LpxL homologue, Vc0213 (Hankins and Trent, 2009), which catalyses the transfer of a myristate (C14:0) residue (Fig. 1B). Notably, phosphorylation of the Kdo sugar was also shown to be required for the function of LpxL homologues from *Haemophlius influenzae* and *Bordetella pertussis* (Hankins and Trent, 2009).

Interestingly, the *V. cholerae* genome encodes an additional secondary acyltransferase homologue annotated as Vc0212. Our present study demonstrates that Vc0212 functions as a novel lipid A secondary acyltransferase responsible for the transfer of a 3-hydroxylaurate (3-OH C12:0) to the acyl chain linked at the 3′-position of *V. cholerae* lipid A (Fig. 1B). Although the primary lipid A acyltransferases, LpxA (Crowell *et al*., 1986; Kelly *et al*., 1993) and LpxD (Kelly *et al*., 1993), catalyse the transfer of β-hydroxyacyl chains from acyl–acyl carrier protein (acyl-ACP) donors to the glucosamine backbone, a lipid A secondary hydroxy-acyltransferase has not been characterized. Given the unique substrate preference, we propose that secondary hydroxy-acyltransferases be given the *lpx* designation, LpxN.

While other Gram-negative bacteria have been shown to possess hydroxylated secondary acyl chains, the hydroxylation typically occurs at the 2-position of the secondary acyl chain following *de novo* synthesis of lipid A. The incorporation of the hydroxyl group is catalysed by LpxO, a membrane bound dioxygenase (Gibbons *et al*., 2000; 2005;). Therefore, our findings reveal a new mechanism for the incorporation of secondary hydroxylated acyl chains to the lipid A of Gram-negative organisms, suggesting that the presence of a hydroxyl group may be evolutionarily important for the stabilization of the outer membrane. Our current data, in combination with our previous work, also clarify the late stages of *V. cholerae* Kdo-lipid A biosynthesis and define the lipid A structure of *V. cholerae* serogroups O1 and O139.

## Results

### *V. cholerae* synthesize a hexa-acylated lipid A species

**Fig. 2:**
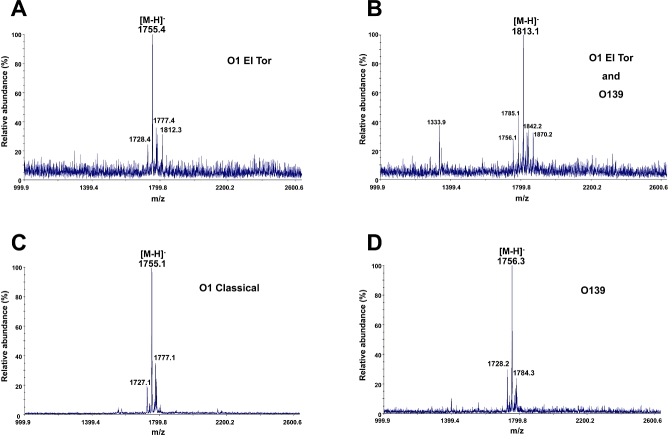
MALDI-TOF mass spectrometry of *V. cholerae* serogroups O1 and O139. Lipid A species of *V. cholerae* O1 El Tor, O1 classical and O139 were isolated and fractionated by DEAE-cellulose chromatography using published protocols (Zhou *et al*., 1999). *V. cholerae* O1 El Tor strains produced lipid A species giving predominant peaks at *m/z* 1755.4 and *m/z* 1813.1, (A) and (B) respectively. *V. cholerae* O139 showed similar results [(D) plus a spectrum similar to the one in (B)]. However, the *V. cholerae* O1 classical strain only produced a predominant peak at *m/z* 1755.1 (C). Although mass spectrometry data were acquired on lipids eluting in all salt fractions from DEAE columns, only the most representative data are shown. Data shown in (A), (C) and (D) are from lipids eluting in the 240 mM fraction; however, lipids eluting in the 120 mM fraction are shown in (B). The minor peaks at *m/z* 1728.4, *m/z* 1785.1, *m/z* 1727.1 and *m/z* 1728.2 arise from a lipid A with an acyl chain reduced by two carbons in length compared with the major ion. The peaks at *m/z* 1813.1 and *m/z* 1812.3 might arise from a lipid A species with extended acyl chains (by 4 carbons). The minor peaks at *m/z* 1842.2 and *m/z* 1784.3 arise from lipid A species with an acyl chain addition of two carbons in length compared with the major ion. Minor peaks at 1777.4 (A) and 1777.1 (C) *m/z* represent sodium adducts of the major parent ion.

To date, no definitive lipid A structure has been determined for *V. cholerae*; however, a previous literature review included proposed lipid A structures for *V. cholerae* O1 and O139 (Chatterjee and Chaudhuri, 2006) (Fig. S1). Although earlier reports characterized the fatty acyl chain composition of various *V. cholerae* strains (Armstrong and Redmond, 1974; Raziuddin and Kawasaki, 1976; Raziuddin, 1977; Broady *et al*., 1981), each investigation failed to examine intact lipid A species of *V. cholerae*. In order to elucidate the intact lipid A structure of *V. cholerae*, lipid A species were isolated from *V. cholerae* serogroups O1 El Tor (E7946 and C6706), O1 classical (O395) and O139 (MO10). Lipid A species were subjected to anion-exchange chromatography as described in *Experimental procedures*. Fractionated lipid A species were analysed by matrix-assisted laser desorption/ionization-time of flight (MALDI-TOF) mass spectrometry (MS) in the negative ion mode (Fig. 2).

**Fig. 3:**
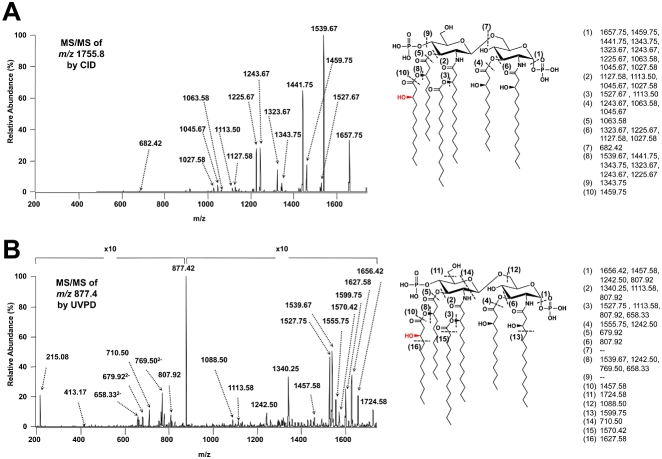
*V. cholerae* synthesize a hexa-acylated lipid A domain. CID (A) and UVPD (B) mass spectrometry were used to analyse the predominant *V. cholerae* peak (1757.1 u). The precursors for CID, 1- and 193 nm UVPD, 2- were *m/z* 1755.8 and *m/z* 877.4 respectively. Cleavages are indicated by dashed lines and are assigned numbers. The *m/z*-values of the resulting fragment ions are grouped next to each cleavage site on the right-hand side. Some ions are listed by multiple cleavage sites to indicate that they arise from multiple consecutive cleavages. The fragmentation patterns supports that *V. cholerae* synthesize a hexa-acylated lipid A structure bearing a myristate (C14:0) at the 2′-position and a 3-hydroxylaurate (3-OH C12:0) at the 3′-position of the glucosamine disaccharide. The ‘× 10’ denotes a section of the spectrum that has been magnified 10 times in order to more easily visualize product ions.

*V. cholerae* serogroups O1 El Tor and O139 displayed more heterogeneity among lipid A species as compared with the O1 classical biotype. *V. cholerae* O1 El Tor strains produced a predominant peak at *m/z* 1755.4, while *V. cholerae* O139 revealed a major peak at *m/z* 1756.3 in fraction A (Fig. 2A and D, respectively). Both *V. cholerae* O1 El Tor and O139 strains displayed a major peak at *m/z* 1813.1 in fraction B (Fig. 2B). MALDI-TOF analysis of the lipid A isolated from the *V. cholerae* O1 classical biotype revealed a predominant peak at *m/z* 1755.1 (Fig. 2C). Because each *V. cholerae* strain examined was found to synthesize a common lipid A species detected at ∼*m/z* 1756, further investigation using collision induced dissociation (CID) and 193 nm ultraviolet photodissociation (UVPD) MS/MS was conducted to elucidate the *V. cholerae* lipid A structure (Fig. 3). Both of these methods are complementary in that each yields unique diagnostic fragmentation patterns, and when used together, form a powerful tool for the structural characterization of lipid A and its modifications (Madsen *et al*., 2011). For example, CID in general yields simple and predictable spectra mostly from C-O cleavages that result in neutral losses of phosphate groups and fatty acid chains. UVPD, in contrast, yields a larger array of product ions including ones arising from C-C cleavages along fatty acid chains as well as cross-ring and inter-ring glucosamine cleavages. The cleavage of C-C bonds adjacent to hydroxyl groups has also been shown to be preferential upon UVPD (Madsen *et al*., 2011), further cementing the utility of this method for the work herein.

Figure 3A shows the CID spectrum of singly deprotonated *V. cholerae* lipid A as well as its associated fragmentation behaviour map, which matches the *m/z*-values of observed fragment ions to specific cleavage sites along the structure. The combination of cleavages at the two phosphate groups, the inter-ring glucosamine that splits the molecule and allows confirmation that the distal side of the molecule contains four acyl chains whereas the proximal side contains only two. The C-O bonds at the 3- and 3′-linked primary acyl chains and 2′- and 3′-linked secondary acyl chains allow assignment of the general structure of *V. cholerae* lipid A. UVPD of the doubly deprotonated species (Fig. 3B) provides additional structural details, including identification of the key position of a hydroxyl group. In particular, the formation of acyl chain ions of *m/z* 215.08 and 413.17 as well as the neutral loss of the aliphatic tail adjacent to the secondary linked 3-OH C12:0 acyl chain (*m/z* 1627.58) suggests the presence of a hydroxyl group on the 3-position of the 3′-linked secondary acyl chain. However, due to the presence of a primary linked 3-OH C12:0 acyl chain in *V. cholerae* lipid A, which is isobaric with the secondary linked 3-OH C12:0, additional experiments described further in the *Results* were necessary to determine the specific location of the hydroxyl modification. Overall, we were able to deduce that *V. cholerae* synthesize a *bis*-phosphorylated hexa-acylated lipid A bearing two acyloxyacyl-linked acyl chains, a myristate (C14:0) at the 2′-position and a probable β-hydroxylaurate (3-OH C12:0) at the 3′-position (Fig. 3). These data further corroborate our previous report which characterized the *V. cholerae* LpxL homologue, Vc0213, as a myristoyl transferase (Hankins and Trent, 2009).

### Vc0212 (LpxN) functions as a lipid A acyltransferase

**Fig. 4:**
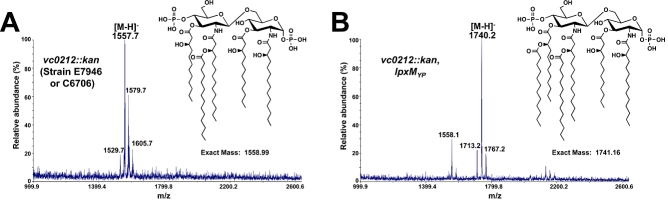
Vc0212 is a *Vibrio* lipid A acyltransferase. A. Lipid A was isolated from E7946 *vc0212::kan* and analysed by MALDI-TOF mass spectrometry. A predominant peak at *m/z* 1557.7 was consistent with the loss of a 3-hydroxylaurate (3-OH C12:0) from the *V. cholerae* O1 El Tor lipid A species. Minor peaks at *m/z* 1579.7 and *m/z* 1605.7 represent sodium adducts of the precursor. The minor peak at *m/z* 1529.7 is consistent with a lipid A species containing an acyl chain reduced by two carbons in length compared with the major species. B. MALDI-TOF analysis of lipid A isolated from of E7946 *vc0212::kan, lpxM_YP_*^+^ resulted in a major peak at *m/z* 1740.2, which is consistent with the addition of laurate (C12:0) to *V. cholerae* lipid A. A small fraction of penta-acylated lipid A is also present; however, analysis of other biological replicates shows only hexa-acylated lipid A. The minor peak at *m/z* 1713.2 arises from a lipid A with an acyl chain reduced by two carbons in length compared with the major ion; however, the peak at *m/z* 1767.2 represents a lipid A with an acyl chain addition of two carbons in length. The predicted structure of the major lipid A species for each strain is shown.

The Clusters of Orthologous Groups database (Tatusov *et al*., 2000) identified three putative *V. cholerae* late acyltransferase homologues: Vc0212, Vc0213 and Vc1577. Although the Clusters of Orthologous Groups database recognized Vc1577 as a putative secondary acyltransferase, blastp results revealed that Vc1577 was an unlikely LpxM homologue (*E*-value 3.9, 22% identity); however, Vc0212 showed 41% identity (*E*-value of < 10^−64^) when compared with the *E. coli* LpxM sequence. Therefore, we hypothesized that Vc0212 functioned as a lipid A secondary acyltransferase. In order to test this hypothesis, a *vc0212* deletion mutant was generated in the *V. cholerae* O1 El Tor strain, E7946. The lipid A of E7946 *vc0212::kan* was isolated and analysed by MALDI-TOF MS, producing a major peak at *m/z* 1557.7 (Fig. 4A), which corresponds to the loss of a 3-hydroxylaurate (3-OH C12:0) residue from the predominant wild-type O1 El Tor lipid A species (Fig. 2A). MS/MS analysis by CID indicated the absence of a 3-hydroxylaurate on the 3′-linked acyl chain (Fig. S2) based on the *m/z*-values of the corresponding products arising from cleavages of the phosphate group, the inter-ring glucosamine, and the C-O bonds along the primary and secondary acyl chains. Due to the simplicity of this lipid A structure, the companion UVPD analysis was not undertaken.

Additionally, the *vc0212* transposon insertion was obtained from a non-redundant transposon insertion library in the *V. cholerae* O1 El Tor strain, C6706 (Cameron *et al*., 2008). MALDI-TOF analysis of lipid A species isolated from the C6706 *vc0212* transposon insertion (Cameron *et al*., 2008) revealed a peak at *m/z* 1558.9 (data not shown), which was similar to the lipid A species produced by E7946 *vc0212::kan* (Fig. 4A). Together, these results are consistent with the loss of 3-hydroxylaurate (3-OH C12:0) from the wild-type *V. cholerae* O1 El Tor lipid A structure (Fig. 2) and indicate that Vc0212 is indeed a functional *V. cholerae* secondary acyltransferase. Notably, complementation of E7946 and C6706 *vc0212* mutations restored the hexa-acylated lipid A phenotype in both O1 El Tor strains (Fig. S3).

### *V. cholerae* LpxN is a novel lipid A secondary acyltransferase

The genome of the Gram-negative bacterium *Salmonella typhimurium* encodes the lipid A modifying enzyme LpxO, which is responsible for modification of the 3′-linked secondary acyl chain with a hydroxyl group at the 2-position. LpxO, which is absent in *E. coli*, functions as a membrane bound Fe^2+^/O_2_/α-ketoglutarate-dependent dioxygenase that modifies lipid A on the cytoplasmic face of the inner membrane (Gibbons *et al*., 2000; 2005;). Although the genome of *V. cholerae* does not encode an LpxO homologue according to blastp results, we wanted to determine if a previously unidentified lipid A dioxygenase was responsible for the addition of the hydroxyl group of the secondary laurate. To address this, *ypO2063* of *Yersinia pestis* was introduced into E7946 *vc0212::kan* using plasmid pBADLpxMYP. The *Yersinia* protein was chosen as it has been previously shown to transfer a lauroyl group (C12:0) to the primary linked fatty acid at 3′-position of lipid A (Rebeil *et al*., 2006). MALDI-TOF MS revealed a major peak at *m/z* 1740.2 (Fig. 4B), which is consistent with the addition of a non-hydroxy laurate to lipid A (predicted [M-H]^-^ at *m/z* 1740.16). These data suggest that the hydroxyl group at the 3-position of the fatty acyl chain does not arise from an LpxO-like enzyme in *V. cholerae.*

**Fig. 5:**
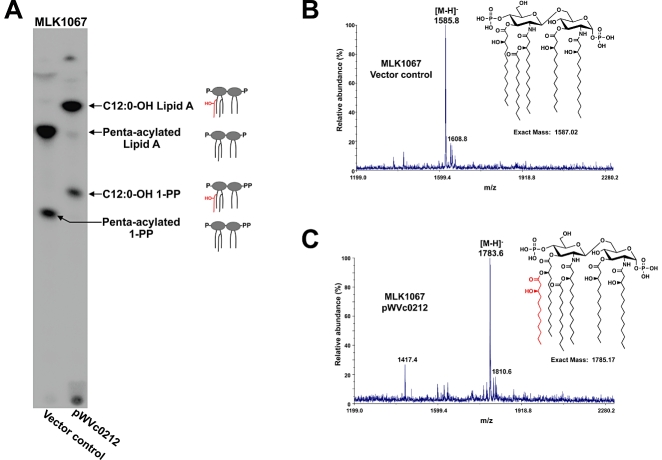
Heterologous expression of Vc0212 in the *E. coli lpxM* deficient strain MLK1067. ^32^P-labelled lipid A species from MLK1067 expressing pWSK29 and pWVc0212 were analysed by TLC and visualized by phosphorimaging (A). Major lipid A species are indicated with arrows. Lipid A species of MLK1067 containing pWSK29 (B) and pWVc0212 (C) were analysed by MALDI-TOF mass spectrometry. The major peak in (B) is *m/z* 1585.8, which corresponds to the expected mass of deprotonated penta-acylated lipid A. The major peak in (C) is *m/z* 1783.6, indicative of the addition of a 3-hydroxylaurate (3-OH C12:0) (red) to the glucosamine disaccharide of the lipid A species. Minor peaks at *m/z* 1608.8 and *m/z* 1810.6 are likely sodium adducts of the lipid A species. The predicted structure of the major lipid A species for each strain is shown.

To confirm that LpxN transfers a 3-hydroxylaurate to the 3′-linked primary acyl chain of *V. cholerae* lipid A, LpxN was expressed in the *E. coli lpxM* deficient strain, MLK1067 (Table 2). The lipid A species of ^32^P_i_-labelled MLK1067 containing the empty vector pWSK29 or pWVc0212 were isolated and separated via thin layer chromatography (TLC). As expected MLK1067/pWSK29 synthesized a penta-acylated lipid A, bearing a single secondary acyl chain at the 2′-position (Karow and Georgopoulos, 1992) whereas expression of LpxN resulted in the production of a faster migrating hexa-acylated lipid A species (Fig. 5A). MALDI-TOF analysis of lipid A isolated from MLK1067/pWSK29 yielded an expected peak at *m/z* 1585.8 (Fig. 5B) indicating *bis*-phosphorylated, penta-acylated lipid A [expected (M-H)^-^ at *m/z* 1586.02]. However, MS analysis of lipid A isolated from MLK1067 expressing LpxN produced a peak at *m/z* 1783.6, a difference of 197.8 Da, thereby demonstrating that LpxN catalyses the transfer of 3-hydroxylaurate to *E. coli* lipid A (Fig. 5C).

**Table 2:** Bacterial strains and plasmids used in this study

Strain or plasmid	Genotype or description	Source or reference
Strains		
*Vibrio cholerae*		
N16961	O1 El Tor biotype	S. Payne
E7946	O1 El Tor biotype	K. Klose
E7946 *vc0212::kan*	E7946 with kanamycin resistance cassette in *vc0212*	This work
E7946 *vc0212::kan, vc0212*^+^	*vc0212::kan*, pBADVc0212	This work
E7946 *vc0212::kan, lpxM_YP_*^+^	*vc0212::kan*, pBADLpxMYP	This work
E7946 *vc0212::kan, lpxM_EC_*^+^	*vc0212::kan*, pBADLpxMEC	This work
C6706	O1 El Tor biotype, lacZ^-^	Cameron *et al*. (2008)
C6706 Δ*vc0212*	C6706 containing transposon insertion in *vc0212*	Cameron *et al*. (2008)
C6706 Δ*vc0212, vc0212^+^*	C6706 Δ*vc0212*, pBADVc0212	This work
O395	O1 Classical biotype	K. Klose
MO10	Wild-type O139	K. Klose
*Escherichia coli*		
JM109	*end*A1, *rec*A1, *gyr*A96, *thi, hsd*R17 (r_k_–, m_k_+), *rel*A1, *sup*E44, Δ( *lac-pro*AB), [F′*tra*D36, *pro*AB, *laq*I_q_ZΔM15]	Promega
SM10λpir	*λpir rec*A Kan^r^; host and mobilizing strain for pGP704 derivatives	Miller and Mekalanos, 1988
XL-1 Blue	*recA1 endA1 gyrA96thi-1 hsdR17 supE44 relA1 lac*[F′*proAB lacI^q^*ZΔM15::Tn*10* (Tet^R^)]	Stratagene
W3110	Wild type, F^-^ l^-^ rph-1 INV(*rrnD, rrnE*)1 *rph-1*	*E. coli* Genetic Stock center (Yale)
MLK1067	W3110 *lpxM*::Ω*cam*	Karow and Georgopoulos (1992)
Plasmids		
pET21a	Vector containing a T7 promotor, Amp^r^	Novagen
pVc0212	pET21a containing *vc0212*	Hankins and Trent (2009)
pWSK29	Low-copy-number cloning vector, Amp^r^	Wang and Kushner (1991)
pWVc0212	pWSK29 containing *vc0212*	This work
pGEM-T Easy	Vector containing a T7 promotor, Amp^r^	Promega
pGEMVc0212	pGEM-T Easy containing *vc0212*	This work
pGEMVc0212Kan	pGEM-T Easy containing *vc0212*::*kan*	This work
pUC4K	Vector containing *kan* resistance gene Kan^r^	Pharmacia
pHM5	Suicide vector pGP704 carrying *sacB*; Amp^r^ Suc^S^	Runyen-Janecky *et al*. (1999)
pHM5Vc0212Kan	pHM5 carrying *vc0212*::*kan*	This work
pBAD18	Arabinose-inducible expression vector, Amp^r^	Guzman *et al*. (1995)
pBADVc0212	pBAD18 containing *vc0212*	This work
pBADLpxMYP	pBAD18 containing *Y. pestis lpxM* (*ypO2063)*	This work

**Fig. 6:**
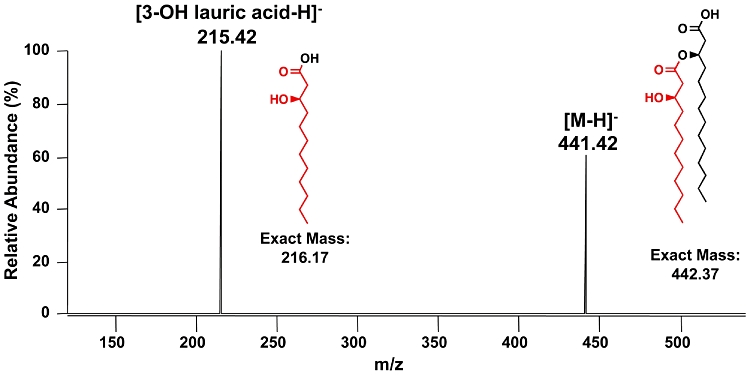
MS^3^ spectrum of lipid A isolated from *E. coli* MLK1067 expressing Vc0212. The doubly deprotonated lipid A ion (*m/z* 891.5) from MLK1067 expressing Vc0212 was subjected to 193 nm UVPD (see Fig. S4) followed by CID of the fragment ion of *m/z* 441.4. The ion of *m/z* 441.4 is interpreted as the deprotonated intact 3′-acyloxyacyl moiety of lipid A (exact mass calculated to be 442.37 u, proposed structure shown in the spectrum). The MS^3^ fragment ion of *m/z* 215.42 is interpreted as deprotonated 3-hydroxylauric acid (exact mass 216.17 u, proposed structure shown in the spectrum).

To confirm this lipid A structure, CID was performed on the singly deprotonated ion, and UVPD was performed on the doubly deprotonated ion as illustrated in Fig. S4. CID again promoted C-O cleavages along the primary and secondary acyl chains, at the phosphate groups, and between the two glucosamines (Fig. S4A). The general structure could be deduced from the CID spectrum alone; however, the exact site of the hydroxylation could not be pinpointed. Figure S4B illustrates the UVPD spectrum and its associated fragmentation map. The additional cleavages of C-C bonds along the fatty acid chain as well as cross-ring and inter-ring glucosamine cleavages are consistent with the structure shown in Fig. S4B. More importantly, the UVPD spectrum confirmed that the hydroxylation occurs at the 3-position of the 3′-linked secondary acyl chain based on the pattern of preferential C-C cleavage adjacent to the hydroxylation. This cleavage denoted as (10) results in the neutral loss of the C9 aliphatic chain, which produces an ion (*m/z* 1655.67) that is not isobaric with any other expected ion. Furthermore, the product ion of *m/z* 441.25 (deprotonated 3′ acyl chain) was subjected to CID (MS^3^) and yielded the fragment ion of *m/z* 215.42 corresponding to the deprotonated 3-OH lauric acid (Fig. 6).

**Fig. 7:**
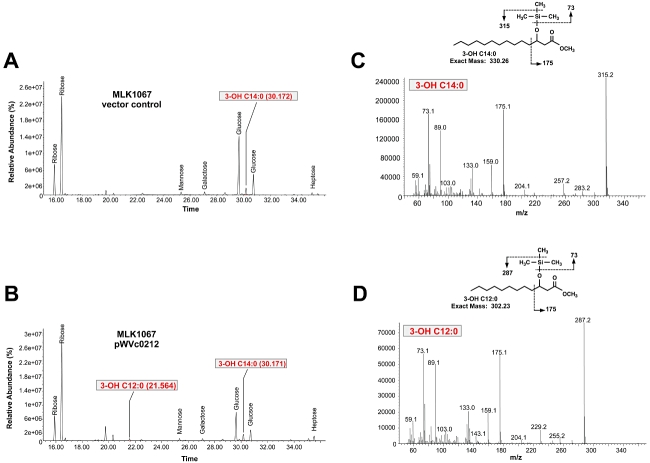
Gas chromatography/mass spectrometry (GC/MS) analysis of hydroxylated fatty acids from LPS isolated from *E. coli* MLK1067 expressing Vc0212. LPS was purified from strain MLK1067, hydrolysed, and converted to TMS derivatives to generate fatty acid methyl esters. (A) shows the total ion chromatogram (TIC) of the GC/MS analysis of hydroxy fatty acids from MLK1067 containing the vector control showing the presence of TMS-3-hydroxymyristoylmethylester (retention time: 30.172). (B) shows the TIC of the GC/MS analysis for MLK1067 containing pWVc0212 showing the presence of both TMS-3-hydroxymyristoylmethylester (retention time: 30.171) and TMS-3-hydroxylauroylmethylester (retention time: 21.564). (C) and (D) show the EI mass spectra of peaks at 30.171 and 21.564 found in the TIC of MLK1067/pWVc0212. Based upon fragmentation patterns of TMS derivatives of bacterial acid methylester standards (Fig. S5), electron ionization/mass spectrometry analysis of the 30.171 peak was indicative of a TMS-3-hydroxymyristoylmethylester (C) and analysis of the 21.564 peak indicative of TMS-3-lauroylmethylester (D). The EI mass spectra of the peak at 30.172 found in the TIC of MLK1067 vector control (A) was identical to that shown in (C). The key cleavages, indicated by dashed lines, are shown for each inserted structure. Sugars present in the sample are also indicated.

Gas chromatography/mass spectrometry (GC/MS) analysis of the trimethylsilylated (TMS) acyl chains from whole LPS isolated from MLK1067 containing either pWSK29 or pVc0212 also showed the location of the hydroxyl group to reside at the 3-position of the laurate (Fig. 7). Based upon the retention times of the standards (Fig. S5), the esters obtained upon methanolysis and silylation of the acyl chains of LPS from MLK1067/pWSK29 corresponded to 3-hydroxymyristoylmethylester (Fig. 7A), whereas LPS from MLK1067 expressing LpxN (Vc0212) yielded the corresponding 3-hydroxylaurate and 3-hydroxymyristate products (Fig. 7B). Electron ionization (EI)/mass spectrometry of TMS derivatives from *E. coli* expressing LpxN produced fragmentation patterns showing the hydroxyl group at the 3-position of both myristoyl (Fig. 7C) and lauroylmethylesters (Fig. 7D). The EI mass spectra of the trimethylsilylated 2-hydroxylauroylmethylester and 2-hydroxymyristoylmethylester standards displayed different fragmentation patterns as compared with the corresponding 3-hydroxy derivatives (Fig. S5). Because wild-type *E. coli* lipid A does not normally contain 3-hydroxylaurate, these results confirm not only the basic framework of the lipid A species upon expression of LpxN, but also the exact site of the hydroxylation on the 3-position of the secondary acyl chain.

### Hydroxylation of the lipid A secondary acyl chain is required for polymyxin B resistance of *V. cholerae*

Polymyxin B is a cationic antimicrobial peptide (CAMP) that binds to the lipid A anchor of LPS, leading to the disruption of the Gram-negative bacterial membrane. Several Gram-negative bacteria possess lipid A modifying enzymes, which aid in survival against CAMPs (Trent *et al*., 2006). *V. cholerae* serogroups O1 and O139 have been shown to be resistant to polymyxin B (Gangarosa *et al*., 1967; Higa *et al*., 1993), indicating that *V. cholerae* may have evolved a lipid A structure to promote CAMP resistance.

To determine if the presence of the 3-hydroxylaurate (3-OH C12:0) residue on the *V. cholerae* lipid A structure was important for the organism's defence against CAMPs, the minimum inhibitory concentration (MIC) of polymyxin B was determined as described in *Experimental procedures*. As shown in Table 1, the loss of Vc0212 (LpxN) activity caused a 50-fold decrease in polymyxin resistance when compared with wild-type levels, indicating that the presence of a 3-hydroxylaurate (3-OH C12:0) was required for polymyxin B resistance in *V. cholerae*. Complementation of E7946 *vc0212::kan* with *vc0212*, restored polymyxin B resistance to wild-type levels. Although expression of YpO2063 restored hexa-acylation of *Vibrio* lipid A (Fig. 4B), it did not restore antimicrobial peptide resistance (Table 1). This finding suggests that the 3-hydroxyl group on the 3′-linked secondary acyl chain is required for polymyxin resistance in *V. cholerae*.

**Table 1:** Polymyxin minimal inhibitory concentration (MIC) of *V. cholerae* strains

Strain	Polymyxin B MIC (µg ml^−1^)^a^
E7946	50.0
E7946 *vc0212::kan*	1.0
E7946 *vc0212::kan, vc0212*^+^	50.0
E7946 *vc0212::kan, lpxM_YP_^+^*	1.0

a MIC values were identical for three biological replicates.

### Hexa-acylation of Vibrio LPS is required for stimulation of TLR4

**Fig. 8:**
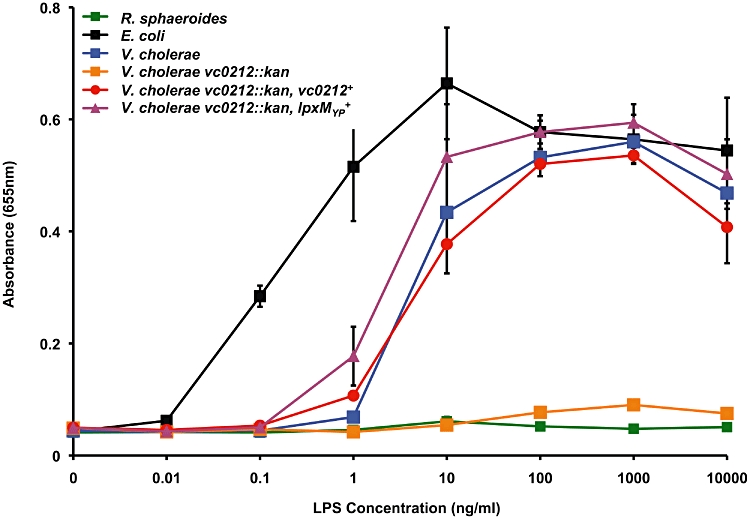
LPS from *vc0212* deletion mutant does not stimulate innate immune recognition by human TLR4-MD2. HEK-293 cells transfected with hTLR4-MD2 and co-receptor hCD14 were treated overnight using 10-fold dilutions of highly purified LPS isolated from the indicated strains of *V. cholerae*. Additionally, LPS from *R. sphaeroides* (green line) and *E. coli* (black line) was utilized as negative and positive controls, respectively. Values are the mean of results from triplicate wells ± standard deviation. LPS isolated from the wild-type *V. cholerae* O1 El Tor (blue line) was found to stimulate TLR4-dependent NF-κB activation in HEK cells, similarly to the positive control (black line). However, LPS isolated from the *vc0212* deletion mutant (orange line) did not elicit TLR4-dependent NF-κB activation. LPS isolated from the *vc0212* deletion mutant complemented with either *vc0212* (red line) or the *Y. pestis* acyltransferase (magenta line) fully restored TLR-4 stimulation of NF-κB. Wild-type LPS and LPS isolated from complemented strains at 1.0–1000 ng ml^−1^ showed a significant increase (*P* ≤ 0.006) in hTLR4-MD2 activation of NF-κB when compared with LPS isolated from the *vc0212* deletion mutant. This figure is available in colour online at http://wileyonlinelibrary.com.

In order to determine if *V. cholerae* LPS possesses immuno-stimulatory properties, LPS was purified using the Hirschfield method (Hirschfield *et al.*, 2000) that allows for removal of potential contaminating lipoproteins. Activation of TLRs was monitored using HEK-Blue 293 cells stably transfected with TLR machinery and a secreted embryonic alkaline phosphatase (SEAP) reporter gene placed under the control of an NF-κB inducible promoter allowing for easy detection of TLR activation using a colorimetric assay. As shown in Fig. 8, wild-type *V. cholerae* LPS causes a potent activation of hTLR4-dependent NF-κB activation; however, LPS isolated from the *vc0212* deletion mutant with a penta-acylated lipid A (Fig. 4A) is attenuated when assayed for hTLR4 activation. Interestingly, when the *vc0212* deletion mutant was complemented with a plasmid expressing either LpxN (Vc0212) or the *Yersinia* enzyme, hTLR4-dependent NF-κB stimulation returned to wild-type levels (Fig. 8). When comparing hTLR4 activation using LPS isolated from the *vc0212* deletion mutant with LPS isolated from wild type or complemented strains at concentrations between 1.0 and 1000 ng ml^−1^, hTLR4-dependent NF-κB activation was significantly reduced (*P* ≤ 0.006).

Within the literature there are reports of Gram-negative organisms producing an LPS structure that activates human TLR2 (hTLR2). Examples include LPS from *Porphyromonas gingivalis* (Darveau *et al*., 2004; Hashimoto *et al*., 2004; Que-Gewirth *et al*., 2004; Ogawa *et al*., 2007) and *Leptospira interrogans* (Werts *et al*., 2001). hTLR2 also recognizes other conserved bacterial structures including lipoteichoic acid and lipoproteins (Hashimoto *et al*., 2004; Ogawa *et al*., 2007). LPS from *V. cholerae* did not activate HEK293 cells transfected with hTLR2 (Fig. S6). Altogether, these results indicate that the presence of an acyl chain linked at the 3′-position is required for the stimulation of the innate immune system via hTLR4-MD2. However, the 3-hydroxyl group does not play a role in hTLR4 modulation. Furthermore, as is the case with the vast majority of Gram-negative organisms, LPS from *V. cholerae* did not stimulate hTLR2.

## Discussion

Although several studies have characterized the fatty acid compositions of *V. cholerae* LPS (Armstrong and Redmond, 1974; Raziuddin and Kawasaki, 1976; Raziuddin, 1977; Broady *et al*., 1981; Kabir, 1982), these findings did not provide conclusive evidence for the proposed *V. cholerae* Kdo-lipid A structures previously reported by Chatterjee and Chaudhuri (2006) (Fig. S1) (Chatterjee and Chaudhuri, 2006). *V. cholerae* serogroup O1 was proposed to possess a symmetrical, hexa-acylated Kdo-lipid A domain, bearing secondary acyl chains at the 2- and 2′-positions; however, *V. cholerae* O139 was reported as having an octa-acylated Kdo-lipid A domain (Fig. S1). The Kdo-lipid A biosynthetic pathway of *V. cholerae* had received little attention until a previous report from our laboratory investigated the late stages of *V. cholerae* Kdo-lipid A biosynthesis (Fig. 1B) (Hankins and Trent, 2009). Phosphorylation of the *V. cholerae* Kdo sugar was shown to be required for the functionality of the *V. cholerae* secondary acyltransferase, Vc0213 (Fig. 1B). Vc0213 acts as the *V. cholerae* LpxL homologue, transferring a myristate (C14:0) to the phosphorylated Kdo-lipid A domain of *V. cholerae*. Our findings herein identify the novel lipid A secondary acyltransferase LpxN, encoded by *vc0212*, which catalyses the transfer of 3-hydroxylaurate (3-OH C12:0) to the 3′-linked acyl chain of *V. cholerae* lipid A. Additionally, MS analysis of intact lipid A have provided a definitive structure of a major lipid A species found on the surface of *V. cholerae* serogroups responsible for human disease.

Mass spectrometry analyses elucidated the predominant lipid A species of four *V. cholerae* strains associated with cholera outbreaks: C6706, E7946, MO10 and O395 (Figs 2 and 3). Our findings demonstrated that *V. cholerae* synthesize a β, 1′-6 linked glucosamine disaccharide with unmodified 1- and 4′-phosphate groups, which is acylated at the 2-, 3-, 2′- and 3′-positions. Myristate (C14:0) and 3-hydroxylaurate (3-OH C12:0) are ester-linked to the hydroxyl groups on the 2′- and 3′-linked fatty acyl chains, creating an asymmetrical hexa-acylated lipid A (Figs 2 and 3). These results demonstrate that *V. cholerae* serogroups O1 and O139 synthesize a similar, asymmetrical lipid A species that is different than the lipid A structures reviewed by Chatterjee and Chaudhuri (2006).

LpxN was required for the transfer of a hydroxylated secondary acyl chain to the 3′-linked acyl chain of the glucosamine disaccharide (Fig. 3A). This finding was further confirmed by the heterologous expression of LpxN in the *E. coli lpxM* mutant, MLK1067 (Figs 5–7). Although lipid A secondary acyltransferases have been well characterized in many Gram-negative bacteria, this is the first report to demonstrate the addition of a hydroxyl group to the secondary acyl chain independent of LpxO.

A recent report found that *V. cholerae vc0212* aids in resistance to antimicrobial peptides, including polymyxin B and LL-37 (Matson *et al*., 2010). However, MS analysis reported by Matson and coworkers (2010) demonstrated a hexa-acylated lipid A peak at *m/z* 1766.165 and a penta-acylated peak at *m/z* 1539.971, which is inconsistent given our current findings. These data were also inconsistent with either of the proposed *V. cholerae* lipid A structures for serogroups O1 and O139 (Fig. S1) and the proposed structure by Matson and coworkers (2010). The authors also reported a symmetrical lipid A structure for serogroup O1 with a secondary acyl chain linked at the 2- and 2′-positions. However, we have clearly demonstrated that *V. cholerae* lipid A is asymmetrical with regard to its acylation pattern (see structure in Fig. 1). Furthermore, Matson *et al*. (2010) did not report the incorporation of a secondary 3-hydroxylated fatty acyl chain, a unique feature of *V. cholerae* LPS. Given that complementation of the *vc0212* mutant with the *Yersinia* lauroyl transferase does not restore polymyxin resistance (Table 1), the incorporation of the 3-hydroxyl group provides a competitive advantage for *V. cholerae* in the presence of CAMPs.

Interestingly, LPS isolated from *V. cholerae* strains C6706 (El Tor) and O395 (classical) *vc0212* mutants previously failed to stimulate hTLR4 (Matson *et al*., 2010); however, complementation studies were not previously reported. Here, we confirm in the El Tor strain E7946 that loss of hexa-acylation of *V. cholerae* LPS results in a reduction in hTLR4 activation. Furthermore, through complementation studies, we also demonstrate that hTLR4 activation of the *vc0212* deletion mutant can be restored to wild-type levels when complemented *in trans* with either *V. cholerae* or *Y. pestis* acyltransferases (Fig. 8). These results indicate that the 3-hydroxyl group on the 3′-linked acyl chain plays a differential role in antimicrobial peptide resistance as compared with hTLR4 stimulation.

Other Gram-negative bacteria have been shown to synthesize lipid A species that possess a hydroxyl group on a secondary acyl chain. The lipid A structures of *Salmonella* (Gibbons *et al*., 2000; 2005;), *Pseudomonas* (Kulshin *et al*., 1991) and *Legionella* (Zahringer *et al*., 1995) contain 2-hydroxylations on the lipid A secondary acyl chains and an LpxO homologue is encoded in each organism's genome. Although *V. cholerae* does not contain an LpxO homologue, the organism has evolved an alternative mechanism to obtain a hydroxylation on the lipid A secondary acyl chain. Presumably, by utilizing β-hydroxylated fatty acid donors (e.g. acyl-acyl carrier proteins), Vc0212 can catalyse the transfer of a 3-hydroxylaurate (3-OH C12:0) to the lipid A domain of *V. cholerae*.

Interestingly, the lipid A species of the aquatic Gram-negative bacteria, *Marinomonas communis* (Vorob'eva *et al*., 2005) and *Marinomonas vaga* (Krasikova *et al*., 2004), were also reported to synthesize lipid A species containing hydroxylated secondary acyl chains. A recent report on the lipid A structure of *Vibrio fischeri* showed a secondary 3-hydroxymyristate (3-OH C14:0) at the 2′-position (Phillips *et al*., 2011). A blastp search did not reveal any LpxO homologues in the genomes of *M. communis, M. vaga* or *V. fischeri* similarly to *V. cholerae*. Thus, the incorporation of a hydroxyl group to the lipid A of these Gram-negative bacteria may occur by an LpxN hydroxy-acyltransferase. The presence of a lipid A secondary hydroxy-acyltransferase may also represent an important evolutionary link among aquatic Gram-negative bacteria and may play a key role in environmental or host survival.

These results together with our previous study (Hankins and Trent, 2009) clarify the late stages of *V. cholerae* Kdo-lipid A biosynthesis (Fig. 1B) and elucidate the *V. cholerae* lipid A structure. Additionally, we have identified a lipid A secondary acyltransferase, which promotes polymyxin B resistance of *V. cholerae*. Although it is unclear how the 3-hydroxyl group aids *V. cholerae* in resistance to polymyxin B, Nikaido and coworkers (Nikaido, 2003; Murata *et al*., 2007) have hypothesized that LpxO activity may increase hydrogen bonding between LPS molecules, thereby decreasing outer membrane permeability. Further, the presence of a 3-hydroxyl group on the secondary acyl chain may provide a site for esterification of additional functional groups. This is currently under investigation in our laboratory.

## Experimental procedures

### Bacterial strains and growth conditions

The bacterial strains and plasmids used in this study are summarized in Table 2. *V. cholerae* strains were grown routinely at 37°C in Luria–Bertani (LB) broth or on LB agar or in a modified g56 minimal media containing 45 mM HEPES, pH 7.5, 10 mM KCl, 10 mM (NH_4_)_2_SO_4_, 0.2% glucose, 1 mM MgSO_4_, 1 mM CaCl_2_, 0.015 mM FeSO_4_, 0.075 mM thiamine and 0.15 mM KH_2_PO_4_ (Galloway and Raetz, 1990). Typically, *E. coli* strains were grown in LB at 37°C unless indicated otherwise. Antibiotics were used at the following concentrations: 100 µg ml^−1^ ampicillin, 60 µg ml^−1^ kanamycin, 10 µg ml^−1^ streptomycin. For *V. cholerae* complementation, 0.2% L-arabinose (Acros Organics) was added to growth media.

### Recombinant DNA techniques

Plasmids were isolated using the QIAprep Spin Miniprep Kit (Qiagen). Custom primers were obtained from Integrated DNA Technologies (Table S1). Polymerase chain reaction (PCR) reagents were purchased from Takara, New England Biolabs or Stratagene and PCR products were isolated using QIAquick PCR Purification Kit (Qiagen). DNA fragments were isolated from agarose gels using the QIAquick Gel Extraction Kit (Qiagen). All other modifying enzymes were purchased from NEB and were used according to manufacturers' instructions.

### Construction of vc0212 defective mutant in *V. cholerae* O1 El Tor strain, E7946

*Vc0212* (*lpxN*) and 1000 bp flanking sequence upstream and downstream were amplified by PCR from N16961 genomic DNA using primers Fvc02121kb and Rvc02121kb (Table S1). The amplicon was ligated into pGEM-T Easy (Promega), creating pGEMVc0212. The vector, pGEMVc0212 was used as template for inverse PCR (primers Fvc0212iPCR and Rvc0212iPCR), which removed 670 bp from *vc0212* and inserted the restriction sites AvrII and AscI into the gene of interest. The kanamycin resistance cassette (kan) from pUC4K was obtained by PCR (KanF and 212KanR) and was inserted into the AvrII and AscI sites of the inverse PCR product, creating pGEMVc0212Kan. PCR was done to amplify the *vc0212::kan* and the 1000 bp flanking sequence (primers Fvc0212pHM5 and Rvc0212pHM5) and the amplicon was digested with BglII and ligated into the *λpir*-dependent suicide vector, pHM5, creating pHM5Vc0212Kan. pHM5Vc0212Kan was conjugated from SM10*λpir* into E7946 as described previously (Mey *et al*., 2005).

### Complementation of E7946 and C6706 *vc0212* defective mutants

The coding sequence of *vc0212* (*lpxN*), including 20 nucleotides upstream of the *vc0212* start codon, was PCR amplified using primers Fvc0212pBAD and Rvc0212pBAD (Table S1). The amplicon was digested with XbaI and SalI and inserted into the arabinose inducible plasmid, pBAD18, creating pBADVc0212. The plasmid pBADVc0212 was then electroporated into the *vc0212* mutants. The E7946 and C6706 *vc0212* mutants were also complemented with pBADLpxM_EC_ and pBADLpxM_YP_, which were cloned as described above using primers found in Table S1.

### Cloning *vc0212* into the low-copy vector pWSK29

The *vc0212* (*lpxN*) coding region, along with the pET21 ribosomal binding site, was excised from pVc0212 (Hankins and Trent, 2009) using XbaI and XhoI. The gene fragment was inserted into the low-copy vector pWSK29 (Wang and Kushner, 1991) to yield pWVc0212. pWVc0212 was transformed into XL-1 Blue for propagation. pWVc0212 was then transformed into W3110 and MLK1067 to be used to isolate lipid A species for radiolabelling and MS.

### Isolation and visualization of ^32^P-labelled lipid A species

Seven millilitre cultures of MLK1067 and W3110 expressing pWVc0212 or pWSK29 were started at an OD_600_ of ∼0.05 in LB at 30°C. Cultures were induced using 0.5 mM isopropyl-β-D-thiogalactopyranoside (IPTG) (Fisher) and labelled using 2.5 µCi ml^−1^^32^P_i_ (Perkin Elmer). Cells were harvested at an OD_600_ of 1.0 by centrifugation and the isolation of ^32^P-labelled lipid A carried out by mild acid hydrolysis as previously described (Tran *et al*., 2006). ^32^P-lipid A species (10 000 cpm per lane) were spotted onto a Silica Gel 60 TLC plate (EM Separation Technology) and were separated using a solvent system consisting of chloroform, pyridine, 88% formic acid and water (50:50:16:5, v/v). TLC plates were exposed overnight to a PhosphorImager screen, and ^32^P-labelled lipids were detected and analysed using a Bio-Rad personal molecular imager equipped with Quantity One software.

### Large-scale isolation and MALDI-TOF MS of lipid A

Typically, *V. cholerae* (500 ml) and *E. coli* (200 ml) cultures were grown at 37°C and 30°C, respectively, to an OD_600_ of ∼1.0. Cells were harvested by centrifugation and lipid A was released from cells and purified as previously described (Tran *et al*., 2006). The lipid A species were analysed using a MALDI-TOF/TOF (ABI 4700 Proteomics Analyzer) mass spectrometer in negative and positive ion modes with the linear detector as previously described (Hankins and Trent, 2009). Lipid A species were dissolved in 5 ml of chloroform/methanol/water (2:3:1, v/v) and were applied to a 1 ml DEAE-cellulose (Whatman) column in the acetate form (Raetz and Kennedy, 1973; Raetz *et al*., 1985; Zhou *et al*., 1999). Five millilitres of fractions were collected using chloroform/methanol/increasing concentrations of aqueous ammonium acetate (60 mM, 120 mM, 240 mM and 480 mM) (2:3:1, v/v). Each fraction was then converted to two-phase Bligh/Dyer mixtures by adding chloroform and methanol. The lower phases containing the lipid A species were dried under N_2_ and stored at −20°C. The lipid A species were analysed using a MALDI-TOF/TOF (ABI 4700 Proteomics Analyzer) mass spectrometer in negative and positive ion modes with the linear detector as previously described (Hankins and Trent, 2009).

### CID and UVPD MS of lipid A

All mass spectrometric experiments were undertaken on a Thermo Fisher Scientific LTQ XL (San Jose, CA, USA) equipped with a 193 nm Coherent ExciStar XS excimer laser (Santa Clara, CA, USA). The setup was similar to that previously described (Gardner *et al*., 2008; Madsen *et al*., 2010). A *q*-value of 0.1 and UV photoirradiation consisting of five pulses (8 mJ per pulse) at a 500 Hz rep rate (over a 10 ms period) were used for all UVPD experiments. For CID, a *q*-value of 0.25, an activation time of 30 ms, and a normalized collision energy of 35% were used. The maximum injection time for all MS/MS spectra was set to 100 ms. An online nanoelectrospray setup was used for direct infusion with the spray emitter operated at a voltage of 2 kV. Details of the setup have been reported previously (Madsen *et al*., 2011). Dried lipid A samples were reconstituted with 70 µl of 50/50 methanol/chloroform, and were infused at 300 nl min^−1^.

### Polymyxin B MIC assay

*Vibrio cholerae* strains were grown overnight in LB media and were diluted 1:100 in fresh LB. Cells were grown to an OD_600_ of ∼0.5 and used to start fresh LB cultures at an OD_600_ of 0.05 containing increasing concentrations of polymyxin B (1, 2, 5, 10, 50, 100 and 200 µg ml^−1^). Polymyxin B MICs were determined by assessing growth inhibition after 4 h at 37°C.

### LPS isolation and purification

Typically, *V. cholerae* (500 ml) cultures were grown at 37°C to an OD_600_ of ∼1.0. Cells were harvested by centrifugation and lyophilized overnight. LPS was isolated using the phenol-water method as described previously (Westphal and Jann, 1965). LPS samples were then further purified using the method described by Hirschfeld and coworkers (2000) to remove other contaminants (e.g. lipoproteins). Purified LPS preparations were resuspended in endotoxin free water.

### Gas chromatography/mass spectrometry analysis

For identification of the fatty acyl chains of LPS, samples were prepared as follows: ∼1 mg of lyophilized LPS was placed into a glass tube, followed by the addition of 800 µl of 1 M methanolic HCl. The solution was incubated for 16 h at 80°C and the methanolysis product dried down under N_2_. The sample was then per-*O*-trimethylsilylated by treatment with 250 µl Tri-Sil reagent (Pierce) at 80°C for 30 min. After cooling, the sample was dried down followed by the addition of 2 ml of hexane. After filtering the solution through a glass wool packed pasteur pipette, the filtrate was dried down under N_2_ until the volume was about 100 µl, from which 5 µl was injected into an Agilent 7890 GC interfaced to a 5975C MSD mass spectrometer operated in the electron ionization mode. The separation was performed on a Grace EC-1 fused silica capillary column (30 m × 0.25 mm ID). Twenty microlitres of bacterial acid methylesters standard, supplied by Matreya LLC (cat# 1114), was treated with 250 µl Tri-Sil reagent (Pierce) at 80°C for 30 min and further processed and analysed as described for the LPS samples.

### TLR signalling assay

Human epithelial kidney (HEK) 293 cells stably transfected with human TLR4, MD2 and CD14 (HEK-Blue-TLR4) were purchased from InvivoGen. These HEK-Blue clones also stably express secreted embryonic alkaline phosphatase (SEAP) under the control of a promoter inducible by NF-κB and activator protein 1 (AP-1). Thus, stimulation of hTLR4 will result in an amount of extracellular SEAP in the supernatant that is proportional to the level of NF-κB induction. The cells were maintained in standard Dulbecco's modified Eagle's medium (DMEM) with 10% heat-inactivated fetal bovine serum (Gibco) supplemented with 4.5 g l^−1^ glucose, 2 mM L-glutamine, 50 U ml^−1^ penicillin, 50 µg ml^−1^ streptomycin, 100 µg ml^−1^ Normocin (InvivoGen) and 1 × HEK-Blue selection (InvivoGen) in a 5% saturated CO_2_ atmosphere at 37°C.

The induction of hTLR signalling in HEK-Blue-TLR4 cells was assessed by measuring SEAP activity using QUANTI-Blue colorimetric assay (InvivoGen). The assay was performed according to manufacturer's protocols. Briefly, cells were seeded in a 96-well plate in triplicate (2.5 × 10^4^ cells/well) in the presence or absence of 10-fold dilutions of purified LPS. Controls included *Rhodobacter sphaeroides* LPS (hTLR4 antagonist) (InvivoGen) and *E. coli* W3110 LPS (hTLR4 agonist). After 20–24 h incubation, supernatants (20 µl) were transferred to a 96-well plate and incubated for 1 h at 37°C with QUANTI-Blue (180 µl). SEAP activity was measured by reading optical density at 655 nm with a Synergy Mx multi-mode microplate reader (BioTek).

## References

[b1] Armstrong IL, Redmond JW (1974). The fatty acids present in the lipopolysaccharide of *Vibrio cholerae* 569B (Inaba). Biochim Biophys Acta.

[b2] Broady KW, Rietschel ET, Luderitz O (1981). The chemical structure of the lipid A component of lipopolysaccharides from *Vibrio cholerae*. Eur J Biochem.

[b3] Cameron DE, Urbach JM, Mekalanos JJ (2008). A defined transposon mutant library and its use in identifying motility genes in *Vibrio cholerae*. Proc Natl Acad Sci USA.

[b4] Chatterjee SN, Chaudhuri K (2006). Lipopolysaccharides of *Vibrio cholerae*: III. Biological functions. Biochim Biophys Acta.

[b5] Clementz T, Raetz CR (1991). A gene coding for 3-deoxy-D-manno-octulosonic-acid transferase in *Escherichia coli*. Identification, mapping, cloning, and sequencing. J Biol Chem.

[b6] Clementz T, Bednarski JJ, Raetz CR (1996). Function of the htrB high temperature requirement gene of *Escherchia coli* in the acylation of lipid A: HtrB catalyzed incorporation of laurate. J Biol Chem.

[b7] Clementz T, Zhou Z, Raetz CR (1997). Function of the *Escherichia coli* msbB gene, a multicopy suppressor of htrB knockouts, in the acylation of lipid A. Acylation by MsbB follows laurate incorporation by HtrB. J Biol Chem.

[b8] Crowell DN, Anderson MS, Raetz CR (1986). Molecular cloning of the genes for lipid A disaccharide synthase and UDP-N-acetylglucosamine acyltransferase in *Escherichia coli*. J Bacteriol.

[b9] Darveau RP, Pham TT, Lemley K, Reife RA, Bainbridge BW, Coats SR (2004). *Porphyromonas gingivalis* lipopolysaccharide contains multiple lipid A species that functionally interact with both toll-like receptors 2 and 4. Infect Immun.

[b10] Galloway SM, Raetz CR (1990). A mutant of *Escherichia coli* defective in the first step of endotoxin biosynthesis. J Biol Chem.

[b11] Gangarosa EJ, Bennett JV, Boring JR (1967). Differentiation between vibrio cholerae and *Vibrio cholerae* biotype El Tor by the polymyxin B disc test: comparative results with TCBS, Monsur's, Mueller-Hinton and nutrient agar media. Bull World Health Organ.

[b12] Gardner MW, Vasicek LA, Shabbir S, Anslyn EV, Brodbelt JS (2008). Chromogenic cross-linker for the characterization of protein structure by infrared multiphoton dissociation mass spectrometry. Anal Chem.

[b13] Gibbons HS, Lin S, Cotter RJ, Raetz CR (2000). Oxygen requirement for the biosynthesis of the S-2-hydroxymyristate moiety in *Salmonella typhimurium* lipid A. Function of LpxO, A new Fe2+/alpha-ketoglutarate-dependent dioxygenase homologue. J Biol Chem.

[b14] Gibbons HS, Kalb SR, Cotter RJ, Raetz CR (2005). Role of Mg2+ and pH in the modification of Salmonella lipid A after endocytosis by macrophage tumour cells. Mol Microbiol.

[b15] Guzman LM, Belin D, Carson MJ, Beckwith J (1995). Tight regulation, modulation, and high-level expression by vectors containing the arabinose PBAD promoter. J Bacteriol.

[b16] Hankins JV, Trent MS (2009). Secondary acylation of *Vibrio cholerae* lipopolysaccharide requires phosphorylation of Kdo. J Biol Chem.

[b17] Hashimoto M, Asai Y, Ogawa T (2004). Separation and structural analysis of lipoprotein in a lipopolysaccharide preparation from *Porphyromonas gingivalis*. Int Immunol.

[b18] Higa N, Honma Y, Albert MJ, Iwanaga M (1993). Characterization of *Vibrio cholerae* O139 synonym Bengal isolated from patients with cholera-like disease in Bangladesh. Microbiol Immunol.

[b19] Hirschfeld M, Ma Y, Weis JH, Vogel SN, Weis JJ (2000). Cutting Edge: Repurification of Lipopolysaccharide Eliminates Signaling Through Both Human and Murine Toll-Like Receptor 2. J Immunol.

[b20] Kabir S (1982). Characterization of the lipopolysaccharide from *Vibrio cholerae* 395 (Ogawa). Infect Immun.

[b21] Karow M, Georgopoulos C (1992). Isolation and characterization of the *Escherichia coli* msbB gene, a multicopy suppressor of null mutations in the high-temperature requirement gene htrB. J Bacteriol.

[b22] Kelly TM, Stachula SA, Raetz CR, Anderson MS (1993). The firA gene of *Escherichia coli* encodes UDP-3-O-(R-3-hydroxymyristoyl)-glucosamine N-acyltransferase. The third step of endotoxin biosynthesis. J Biol Chem.

[b23] Krasikova IN, Kapustina NV, Isakov VV, Dmitrenok AS, Dmitrenok PS, Gorshkova NM, Solov'eva TF (2004). Detailed structure of lipid A isolated from lipopolysaccharide from the marine proteobacterium *Marinomonas vaga* ATCC 27119. Eur J Biochem.

[b24] Kulshin VA, Zahringer U, Lindner B, Jager KE, Dmitriev BA, Rietschel ET (1991). Structural characterization of the lipid A component of *Pseudomonas aeruginosa* wild-type and rough mutant lipopolysaccharides. Eur J Biochem.

[b25] Madsen JA, Boutz DR, Brodbelt JS (2010). Ultrafast ultraviolet photodissociation at 193 nm and its applicability to proteomic workflows. J Proteome Res.

[b26] Madsen JA, Cullen TW, Trent MS, Brodbelt JS (2011). IR and UV Photodissociation as Analytical Tools for Characterizing Lipid A Structures. Anal Chem.

[b27] Matson JS, Yoo HJ, Hakansson K, Dirita VJ (2010). Polymyxin B resistance in El Tor *Vibrio cholerae* requires lipid acylation catalyzed by MsbB. J Bacteriol.

[b28] Mey AR, Craig SA, Payne SM (2005). Characterization of *Vibrio cholerae* RyhB: the RyhB regulon and role of ryhB in biofilm formation. Infect Immun.

[b29] Miller VL, Mekalanos JJ (1988). A novel suicide vector and its use in construction of insertion mutations: osmoregulation of outer membrane proteins and virulence determinants in Vibrio cholerae requires toxR. J Bacteriol.

[b30] Mooi FR, Bik EM (1997). The evolution of epidemic *Vibrio cholerae* strains. Trends Microbiol.

[b31] Murata T, Tseng W, Guina T, Miller SI, Nikaido H (2007). PhoPQ-mediated regulation produces a more robust permeability barrier in the outer membrane of *Salmonella enterica* serovar typhimurium. J Bacteriol.

[b32] Nikaido H (2003). Molecular basis of bacterial outer membrane permeability revisited. Microbiol Mol Biol Rev.

[b33] Ogawa T, Asai Y, Makimura Y, Tamai R (2007). Chemical structure and immunobiological activity of *Porphyromonas gingivalis* lipid A. Front Biosci.

[b34] Phillips NJ, Adin DM, Stabb EV, McFall-Ngai MJ, Apicella MA, Gibson BW (2011). The lipid A from *Vibrio fischeri* lipopolysaccharide: a unique structure bearing a phosphoglycerol moiety. J Biol Chem.

[b35] Que-Gewirth NL, Ribeiro AA, Kalb SR, Cotter RJ, Bulach DM, Adler B (2004). A methylated phosphate group and four amide-linked acyl chains in leptospira interrogans lipid A. The membrane anchor of an unusual lipopolysaccharide that activates TLR2. J Biol Chem.

[b36] Raetz CR, Kennedy EP (1973). Function of cytidine diphosphate-diglyceride and deoxycytidine diphosphate-diglyceride in the biogenesis of membrane lipids in *Escherichia coli*. J Biol Chem.

[b37] Raetz CR, Whitfield C (2002). Lipopolysaccharide endotoxins. Annu Rev Biochem.

[b38] Raetz CR, Purcell S, Meyer MV, Qureshi N, Takayama K (1985). Isolation and characterization of eight lipid A precursors from a 3-deoxy-D-manno-octylosonic acid-deficient mutant of *Salmonella typhimurium*. J Biol Chem.

[b39] Raetz CR, Reynolds CM, Trent MS, Bishop RE (2007). Lipid A modification systems in gram-negative bacteria. Annu Rev Biochem.

[b40] Raziuddin S (1977). Characterization of lipid A and polysaccharide moieties of the lipopolysaccharides from *Vibrio cholerae*. Biochem J.

[b41] Raziuddin S, Kawasaki T (1976). Biochemical studies on the cell wall lipopolysaccharides (O-antigens) of *Vibrio cholerae* 569 B (Inaba) and El-tor (Inaba). Biochim Biophys Acta.

[b42] Rebeil R, Ernst RK, Jarrett CO, Adams KN, Miller SI, Hinnebusch BJ (2006). Characterization of late acyltransferase genes of *Yersinia pestis* and their role in temperature-dependent lipid A variation. J Bacteriol.

[b43] Runyen-Janecky LJ, Hong M, Payne SM (1999). The virulence plasmid-encoded impCAB operon enhances survival and induced mutagenesis in Shigella flexneri after exposure to UV radiation. Infect Immun.

[b44] Tatusov RL, Galperin MY, Natale DA, Koonin EV (2000). The COG database: a tool for genome-scale analysis of protein functions and evolution. Nucleic Acids Res.

[b45] Tran AX, Whittimore JD, Wyrick PB, McGrath SC, Cotter RJ, Trent MS (2006). The Lipid A 1-Phosphatase of *Helicobacter pylori* Is Required for Resistance to the Antimicrobial Peptide Polymyxin. J Bacteriol.

[b46] Trent MS, Stead CM, Tran AX, Hankins JV (2006). Diversity of endotoxin and its impact on pathogenesis. J Endotoxin Res.

[b47] Vorob'eva EV, Dmitrenok AS, Dmitrenok PS, Isakov VV, Krasikova IN, Solov'eva TF (2005). [The structure of uncommon lipid A from the marine bacterium *Marinomonas communis* ATCC 27118T]. Bioorg Khim.

[b48] Wang RF, Kushner SR (1991). Construction of versatile low-copy-number vectors for cloning, sequencing and gene expression in *Escherichia coli*. Gene.

[b49] Werts C, Tapping RI, Mathison JC, Chuang TH, Kravchenko V, Saint Girons I (2001). Leptospiral lipopolysaccharide activates cells through a TLR2-dependent mechanism. Nat Immunol.

[b50] Westphal O, Jann K, Whistler RL (1965). Bacterial lipopolysaccharides: extraction with phenol-water and further applications of the procedure. Methods in Carbohydrate Chemistry, vol. 5.

[b51] Zahringer U, Knirel YA, Lindner B, Helbig JH, Sonesson A, Marre R, Rietschel ET (1995). The lipopolysaccharide of *Legionella pneumophila* serogroup 1 (strain Philadelphia 1): chemical structure and biological significance. Prog Clin Biol Res.

[b52] Zhou Z, Lin S, Cotter RJ, Raetz CR (1999). Lipid A modifications characteristic of *Salmonella typhimurium* are induced by NH4VO3 in *Escherichia coli* K12. Detection of 4-amino-4-deoxy-L-arabinose, phosphoethanolamine and palmitate. J Biol Chem.

