# Supplementary material for: Elucidation of a novel Vibrio cholerae lipid A secondary hydroxy-acyltransferase and its role in innate immune recognition
Source: Mol Microbiol. 2011 Jul 18;81(5):1313–29. doi: 10.1111/j.1365-2958.2011.07765.x (PMC3178793; doi:10.1111/j.1365-2958.2011.07765.x)
Supplement: Supplementary file 1 [file mmi0081-1313-SD1.pdf]

Supplemental Material for "Elucidation of a novel *Vibrio cholerae* lipid A secondary hydroxy-acyltransferase and its role in innate immune recognition"

Jessica V. Hankins,[1] James A. Madsen,[2] David K. Giles,[3] Brandon M. Childers,[4] Karl E. Klose,[4] Jennifer S. Brodbelt,[2] and M. Stephen Trent[3,5,*]

[1]Department of Biochemistry and Molecular Biology, Georgia Health Sciences University, Augusta, Georgia, 30912; [2]Department of Chemistry and Biochemistry, The University of Texas at Austin, Austin, TX, 78712; [3]Section of Molecular Genetics and Microbiology, The University of Texas at Austin, Austin, TX 78712; [4]South Texas Center for Emerging Infectious Diseases and Department of Biology, The University of Texas at San Antonio, San Antonio, TX 78249; [5]The Institute of Cellular and Molecular Biology, The University of Texas at Austin, Austin, TX 78712, USA.

[*]Corresponding Author: M. S. Trent; E-mail: strent@mail.utexas.edu; Telephone: (512) 232-8371; Fax: (512) 471-7088

Supplemental Figures

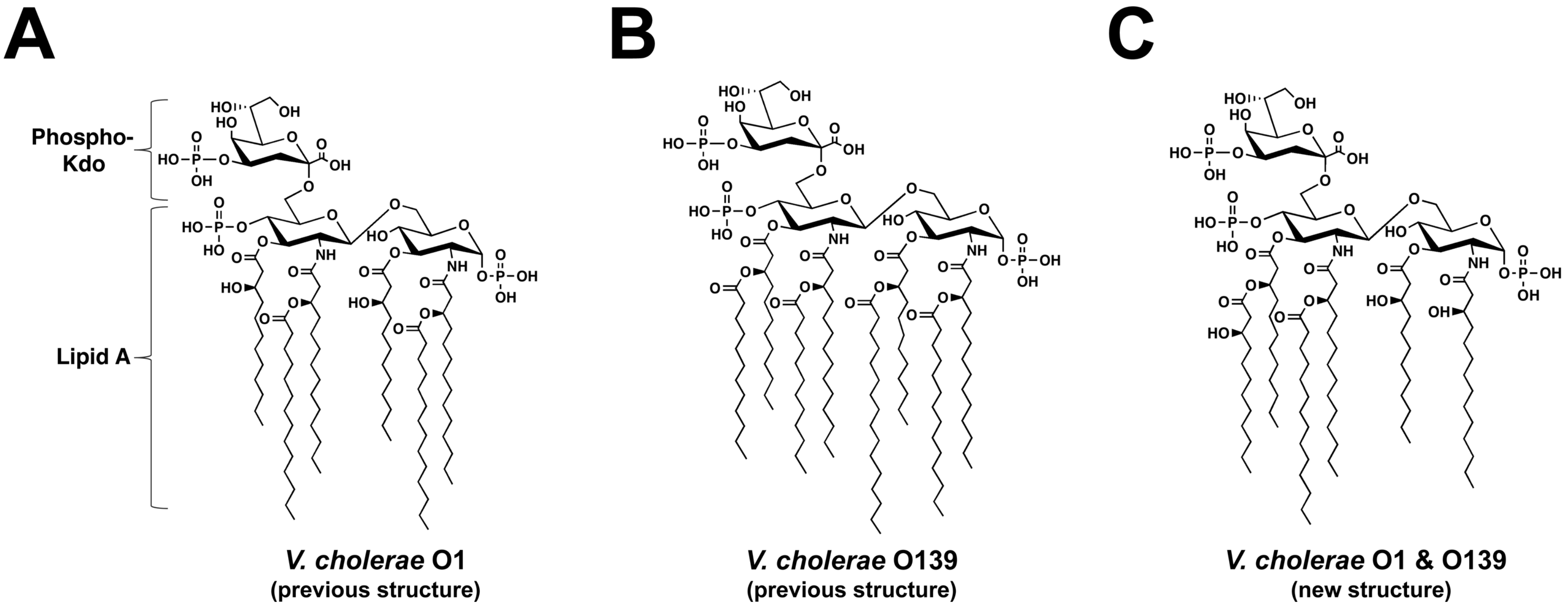

Figure S1. Comparison of the reported structures of *V. cholerae* Kdo-lipid A domains. Panels A and B show previously reported *V. cholerae* Kdo-lipid A structures. The *V. cholerae* serogroup O1 Kdo-lipid A domain was proposed to be hexa-acylated, bearing a myristate (C14:0) at 2- and 2′-positions; however, *V. cholerae* O139 was reported as octa-acylated with all primary linked acyl chains bearing an additional secondary linked fatty acyl chain. Both *V. cholerae* serogroups O1 and O139 were proposed to possess a phosphorylated Kdo sugar, attached to the 6′-position of the glucosamine disaccharide. Additionally a phosphoethanolamine residue (not shown) may be present attached to the 1 or 4′-phosphate groups. Panel C shows the structure of the common lipid A species elucidated in the current work found in both serogroups.

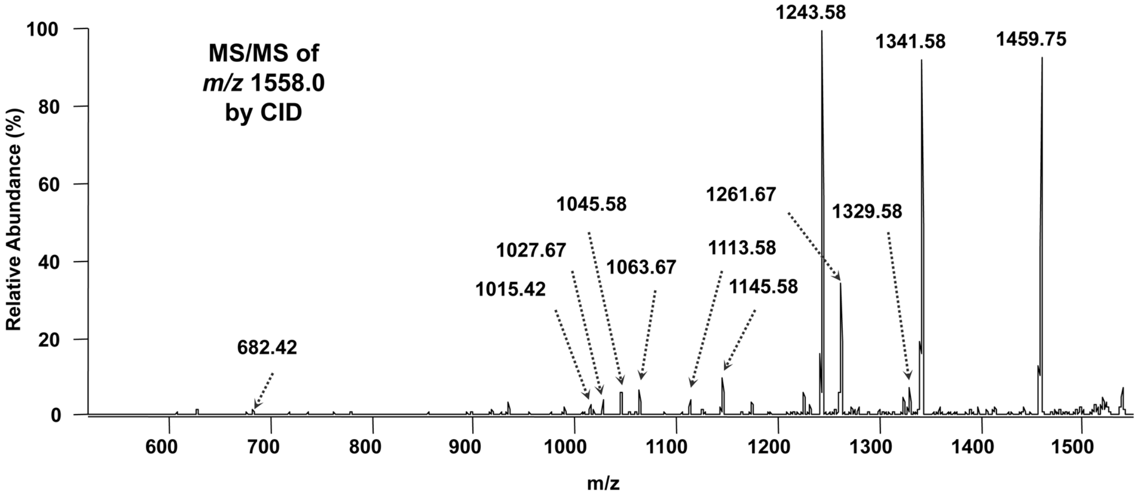

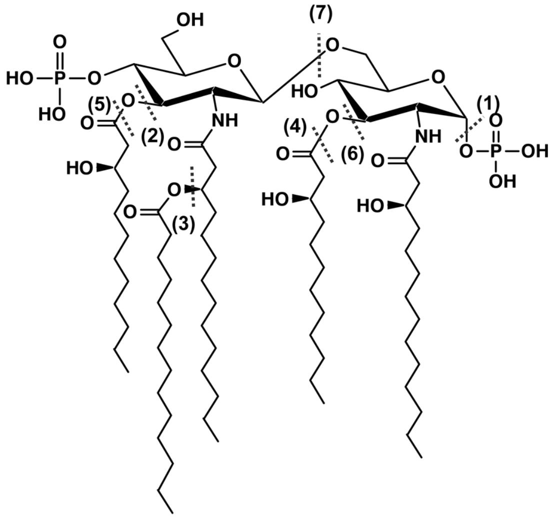

(1) 1459.75, 1261.67, 1243.58, 1063.67, 1045.58, 1027.67, 1015.42
(2) 1341.58, 1243.58, 1145.58, 1113.58, 1045.58, 1027.67, 1015.42
(3) 1329.58, 1113.58, 1015.42
(4) 1261.67, 1145.58, 1063.67, 1045.58
(5) 1063.67
(6) 1027.67
(7) 682.42

Figure S2. MS/MS spectrum from the dissociation of lipid A isolated from E7946 *vc0212::kan* by CID. The $[M-H]^-$ ion was *m/z* 1558.0. Fragmentation behavior is shown and cleavage sites are represented by dashed lines, which are matched with the *m/z* values below the structure.

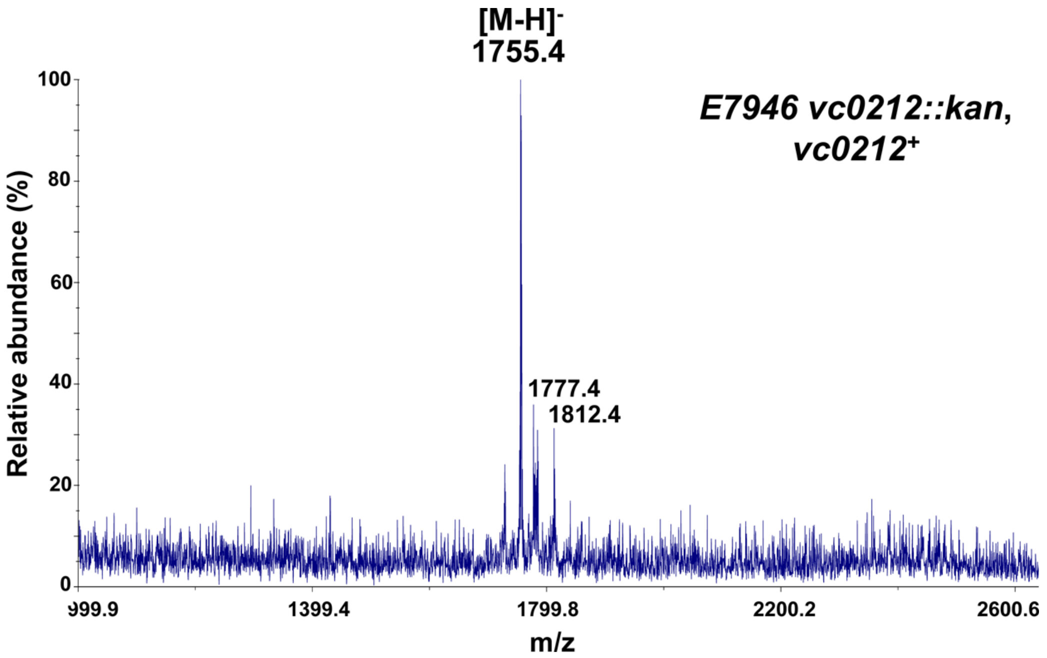

Figure S3. MALDI-TOF mass spectrometry of lipid A from *V. cholerae* E7946 *vc0212::kan, vc0212*$^{+}$*.* Lipid A was isolated from E7946 *vc0212::kan, vc0212*$^{+}$ and analyzed by MALDI-TOF mass spectrometry. A predominant peak at *m/z* 1755.4 was consistent with hexa-acylated lipid A identical to that found in wild type El Tor (see Fig. 2). Similar results were seen with strain C6706 *vc0212::kan, vc0212*$^{+}$.

A

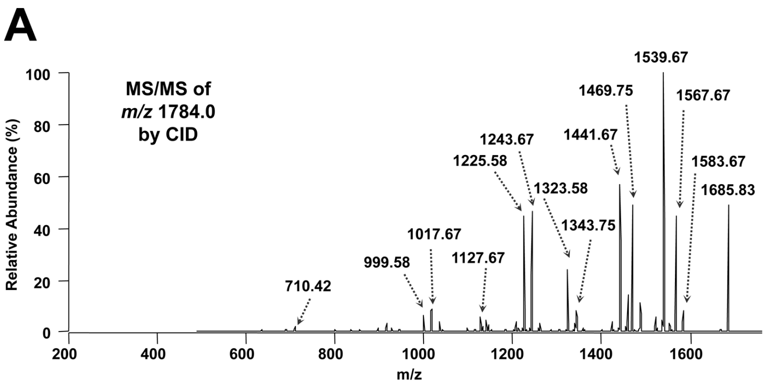

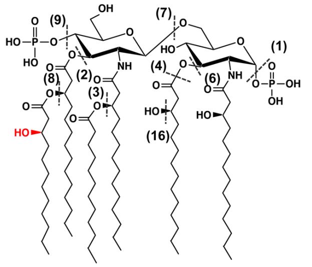

(1) 1685.83, 1469.75, 1441.67, 1243.67, 1225.58, 1127.67, 1017.67, 999.58
(2) 1343.75, 1127.67, 1017.67, 999.58
(3) 1583.67
(4) 1243.67, 1017.67
(5) --
(6) 1539.75, 1441.67, 1323.58, 1225.58, 999.58
(7) 710.42
(8) 1567.67, 1469.75, 1323.58, 1243.67, 1225.58
(9) 1127.67; (16) 1127.67

B

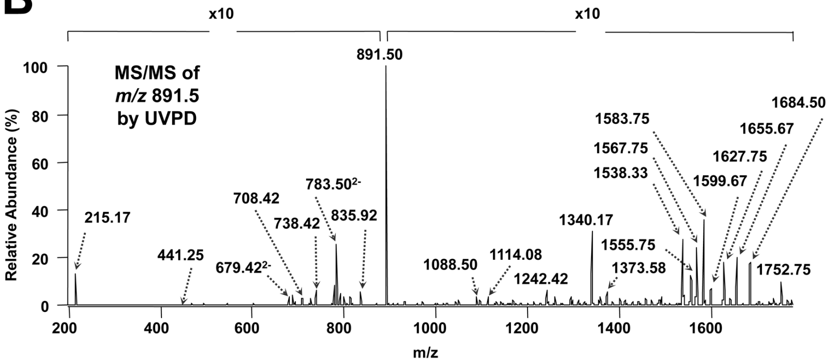

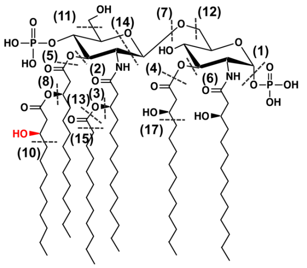

(1) 1684.50, 835.92, 1373.58
(2) 1340.17, 1114.08, 835.92, 441.25
(3) 1583.75
(4) 1555.75, 1242.42, 1114.08, 835.92
(5) 679.42
(6) 1538.33
(7) 708.42
(8) 1567.75, 1242.42, 783.50, 215.17
(9) --
(10) 1655.67, 1373.58; (11) 1752.75, 1373.58
(22) 1088.50; (13) 1599.67, 835.92; (14) 738.42;
(15) 1627.75; (16) --; (17) 1373.58

Figure S4. CID and UVPD spectra from MLK1067 expressing Vc0212. CID, $[M-H]^-$ (A), and 193 nm UVPD, $[M-2H]^{2-}$ (B) mass spectrometry analyses were done on lipid A isolated from MLK1067 expressing Vc0212. The CID, $[M-H]^-$ was *m/z* 1784.0 (A) and the UVPD, $[M-2H]^{2-}$ (B) precursor was *m/z* 891.5. Fragmentation profiles are shown with dashed lines representing cleavage sites and are matched with the *m/z* values below the structure. The "x10" denotes a section of the spectrum that has been magnified ten times in order to more easily visualize product ions.

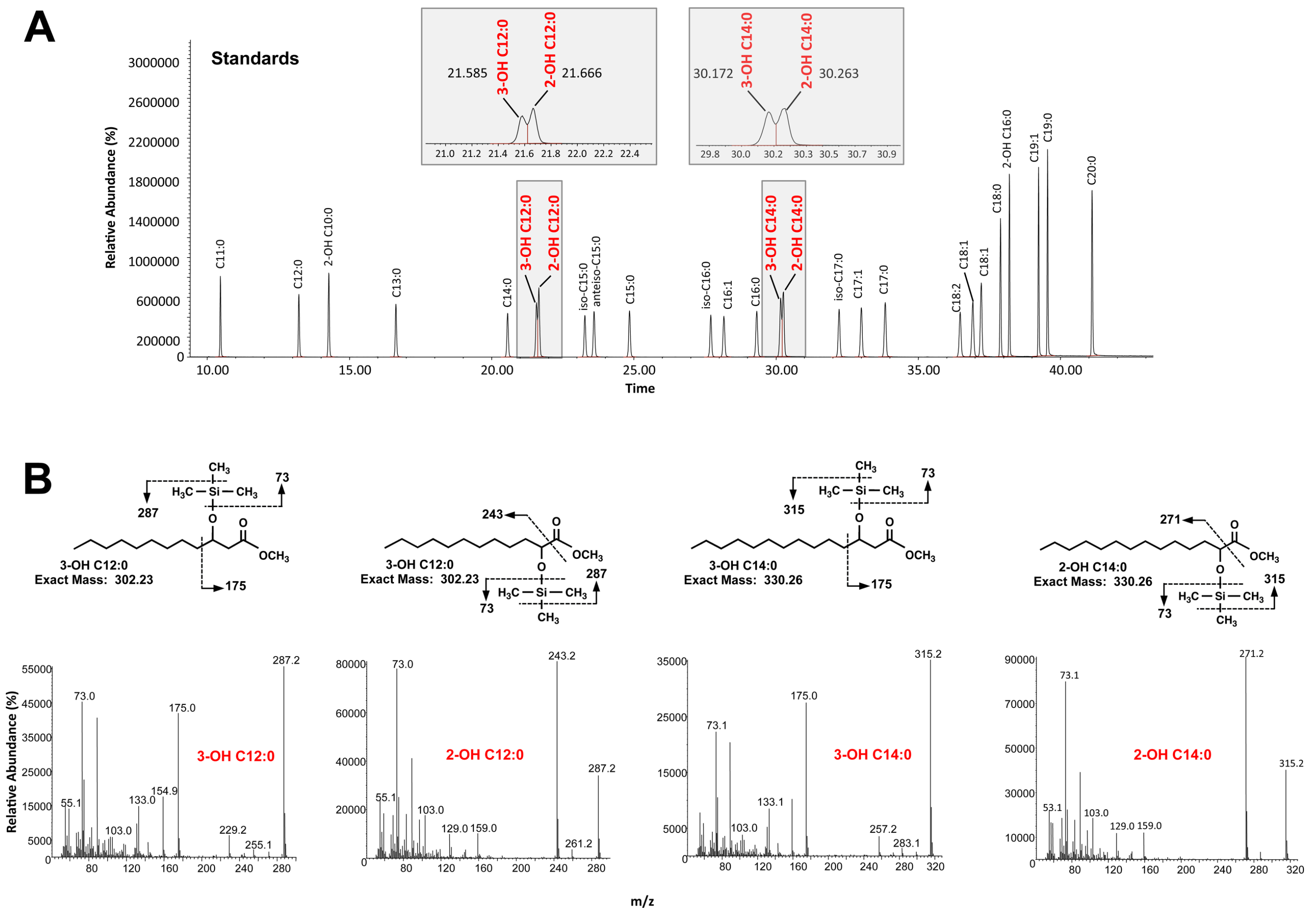

Figure S5. GC/MS analysis of fatty acid standards as TMS derivatives of their methyl esters. Panel A shows the TIC of the GC/MS analysis of bacterial acid methyl esters standard. Panel B shows the EI mass spectra of TMS derivatives of hydroxy fatty acids indicated in Panel A. The key cleavages, indicated by dashed lines, are shown for each inserted structure. Distinct fragmentation patterns were observed for hydroxylated fatty acids depending upon the location of the hydroxyl group (i.e. 2-position versus 3-position). A description of sample preparation and GC/MS conditions are described within the Experimental Procedures section.

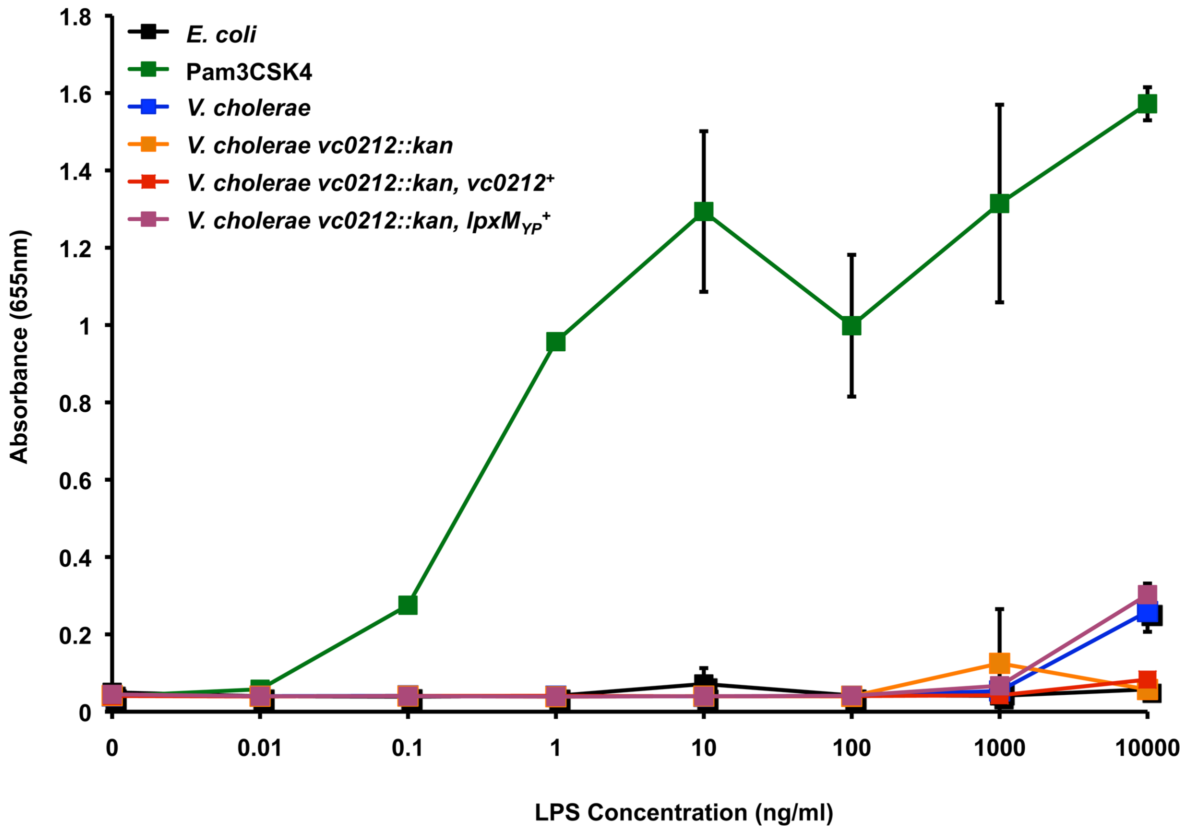

Figure S6. Activation of hTLR2 by *V. cholerae* LPS. Activation of hTLR2 was monitored using HEK-293 cells stably transfected with hTLR2 and 10-fold dilutions of highly purified LPS isolated from the indicated strains of *V. cholerae*. Additionally, LPS from *E. coli* and the synthetic lipopeptide Pam3CSK4 were used as negative and positive controls, respectively. Values are the mean of results from triplicate wells ± standard deviation. Pam3CSK4 was found to stimulate TLR2-dependent NF-κB activation in HEK-cells. However, LPS from either *E. coli* or *V. cholerae* were not stimulatory.

Supplementary Table 1. Oligonucleotides

Name	Sequence
Fvc02121kb	5′-TGAATACTGGGCGCTGCT-3′
Rvc02121kb	5′-AGAGCGATCACACTTAAG -3′
Fvc0212iPCR	5′- GCGCGCGGCGCGCTGCGCTGCAAAACTTCCC-3′
Rvc0212iPCR	5′- GCGCGCCCTAGGCGCAAGCGCCAAGGAATA-3′
KanF	5′-GCGCGCCCTAGGCGAATTCCCCGGATCCGT-3′
212KanR	5′-GCGCGCGGCGCGCCTTAGAAAAACTCATCGAG-3′
Fvc0212pHM5	5′- GCGCGCAGATCTTGAATACTGGGCGCTGCT-3′
Rvc0212pHM5	5′- GCGCGCAGATCTCTTAAGTGTGATCGCTCT-3′
Fvc0212pBAD	5′- GCGCGCTCTAGACTATCAGTAAAGGTGAAT-3′
Rvc0212pBAD	5′- GCGCGCGTCGACTTAGCTTTGATCGCCATGAtg-3′
FLpxMECpBAD	5′- GCGCGCTCTAGATTATCCGAAACTGGAAAA-3′
RLpxMECpBAD	5′- GCGCGCGTCGACTTATTTGATGGGATAAAG-3′
FLpxMYEpBAD	5′- GCGCGCTCTAGACCAATTCTAAGAGTTTCC-3′
RLpxMYEpBAD	5′- GCGCGCGTCGACCTAAAGATCTTTGCGCAC-3′
